# C-953-06. New Skin Cell Suspension Autograft Current Procedural Terminology Codes Impact Payments, Charges and Work Credit

**DOI:** 10.1093/jbcr/irag033.130

**Published:** 2026-04-06

**Authors:** Christopher R LaChapelle, Larissa Epstein, Irma D Fleming, Giavonni Mystique Lewis, Callie M Thompson

**Affiliations:** University of Utah Health, Salt Lake City, UT; University of Utah Health, Salt Lake City, UT; University of Utah Health Burn Center, Salt Lake City, UT; University of Utah Health, Salt Lake City, UT; University of Utah Health, Salt Lake City, UT

## Abstract

**Introduction:**

New current procedural terminology (CPT) codes for skin cell suspension autograft (SCSA) were established following collaboration between the American Burn Association Economics and Access committee and the RVS Update Committee (RUC). These new CPT codes went into effect January 1, 2025. We aim to describe how charges, payments, and work relative value units (wRVU) have been impacted by these new codes.

**Methods:**

This is a single-center retrospective review of all charges, payments, and wRVUs for the SCSA procedure between July 1, 2024, and June 30, 2025, which coincided with 6 months before and 6 months after the new codes were implemented. Internal CPT utilization software was used to query all CPT codes used for SCSA and to identify subjects (Table 1). Only closed accounts which captured payments were included. Demographic and injury data were manually extracted from the electronic medical record. Subjects were divided into one of two groups based on the year the procedure was performed and charged (2024 vs 2025). The primary outcome was payments, and secondary outcomes were charges and wRVUs. Fisher’s exact tests were used to assess categorical variables with small sample sizes. Non paired t-test was used to assess normally distributed continuous variables and Wilcoxon Ranked Sum test was used to assess the non-parametric continuous variables for significance.

**Results:**

One hundred ten SCSA cases were included (n = 77 for 2024, n = 33 for 2025). There were no significant differences between the groups regarding sex, age, insurance, area of SCSA, or operative time. Payments were not significantly different between the 2024 and 2025 groups ($X vs $1.34X, *p*=.802). Charges ($3.66X vs $2.33X, *p*=.012) and wRVUs (34.65 vs 21.28, *p*<.001) both decreased significantly from 2024 to 2025 (Table2).

**Conclusions:**

Payments remained stable with the implementation of the new SCSA CPT codes. There was a notable reduction in wRVUs credited for SCSA procedures despite no significant differences in the area of SCSA and the operative time in the 2 groups. Physician reimbursement will be disproportionately affected in wRVU modeled reimbursement settings. Increasing wRVUs for SCSA to remain commensurate with the work performed and payments received is recommended.

**Applicability of Research to Practice:**

New CPT codes for SCSA affect the financial viability of burn centers and it is important to understand the impact of such changes. Higher wRVUs should be assigned to the SCSA procedures to maintain appropriate physician credit in line with unchanged payments and work.

**Funding for the Study:**

N/A.

